# Recurrence Patterns and Prognostic Factors in Sentinel Lymph Node‐Negative Melanoma in a Taiwanese Cohort With Predominant Acral Melanoma

**DOI:** 10.1111/1346-8138.70358

**Published:** 2026-06-17

**Authors:** Chih‐Yuan Chang, Cheng‐Yuan Li, Tien‐Hsiang Wang, Chi‐Han Tsai, Jai‐Sing Yang, Yu‐Jen Chiu

**Affiliations:** ^1^ Department of Medical Education Taipei Veterans General Hospital Taipei Taiwan; ^2^ Department of Dermatology Taipei Veterans General Hospital Taipei Taiwan; ^3^ Faculty of Medicine, School of Medicine National Yang Ming Chiao Tung University Taipei Taiwan; ^4^ Division of Plastic and Reconstructive Surgery, Department of Surgery Taipei Veterans General Hospital Taipei Taiwan; ^5^ Department of Surgery, School of Medicine National Yang Ming Chiao Tung University Taipei Taiwan; ^6^ Department of Medical Research, China Medical University Hospital China Medical University Taichung Taiwan

**Keywords:** acral melanoma, cutaneous melanoma, prognostic factors, recurrence, sentinel lymph node biopsy

## Abstract

Cutaneous melanoma in Asian populations is characterized by a predominance of acral melanoma and distinct clinicopathologic features. Although sentinel lymph node biopsy (SLNB) is widely used for staging, a subset of SLN‐negative patients still develop recurrence, and evidence regarding recurrence patterns and prognostic factors in this group remains limited, particularly in acral‐predominant cohorts. We conducted a retrospective cohort study of consecutive patients with cutaneous melanoma evaluated at Taipei Veterans General Hospital between 2005 and 2025. A total of 181 patients were included and categorized into SLN‐positive (*n* = 89) and SLN‐negative (*n* = 92) groups for survival analysis, with further analyses of recurrence patterns and prognostic factors restricted to the SLN‐negative cohort. Kaplan–Meier analysis showed significantly poorer recurrence‐free survival (RFS) and overall survival (OS) in the SLN‐positive group compared with the SLN‐negative group (both *p* < 0.001). Among SLN‐negative patients (mean age, 72.3 years; 88.0% acral melanoma), 25.0% developed recurrence during a median follow‐up of 28.5 months, corresponding to a 5‐year recurrence rate of 30.9%. Nodal recurrence was the most frequent initial pattern and tended to occur earlier than other types of recurrence. Multivariable analysis identified lower body mass index, diabetes mellitus, and ulceration as factors associated with poorer RFS, while older age, lower body mass index, and greater Breslow thickness were associated with poorer OS. These findings suggest that recurrence remains a clinically relevant concern in SLN‐negative melanoma in acral‐predominant Asian populations. Careful follow‐up may be warranted, particularly in patients with unfavorable clinicopathologic features.

## 1 Introduction

Cutaneous melanoma is one of the most aggressive forms of skin cancer and a major cause of skin cancer‐related mortality worldwide, representing a significant global health burden [1]. Although the overall incidence of melanoma is lower in Asian populations than in Western countries, the clinicopathologic characteristics and clinical outcomes differ substantially [2].

Cutaneous melanoma (CM) is broadly categorized into four major histological subtypes: lentigo maligna melanoma (LMM), superficial spreading melanoma (SSM), acral lentiginous melanoma (ALM), and nodular melanoma. Histologically, superficial spreading melanoma is the predominant subtype in Western populations, whereas acral lentiginous melanoma (ALM) is the most common histological subtype in Asian populations [2].

Clinically, CM may also be classified anatomically according to whether the tumor arises in acral sites. Acral melanoma (AM), defined by melanoma arising on glabrous skin of the palms, soles, and nail apparatus, represents a disproportionately high proportion of melanoma cases in Asian populations. Several studies have suggested that AM is associated with poorer stage‐matched survival outcomes and distinct recurrence patterns compared with non‐acral melanoma [3, 4].

AM frequently presents with a longer prediagnosis duration with thicker tumors and more advanced disease at diagnosis. In addition to clinicopathologic differences, melanoma in Asian populations also exhibits distinct genomic profiles compared with Western populations. Acral melanoma is characterized by a lower prevalence of BRAF mutations and a higher frequency of structural variations and KIT alterations, suggesting a biologically distinct disease entity [5, 6, 7, 8, 9, 10]. These differences may partly contribute to variations in disease behavior, recurrence patterns, and treatment response observed between Asian and Western melanoma cohorts.

Accurate staging is essential for treatment planning and prognosis in melanoma. The American Joint Committee on Cancer (AJCC) 8th edition staging system incorporates sentinel lymph node biopsy (SLNB) as the standard staging procedure for clinically node‐negative melanoma [11]. Patients with a positive SLN result are upstaged to stage III disease, and SLN positivity is a well‐established adverse prognostic factor. Patients with SLN‐positive melanoma have a significantly higher risk of recurrence and mortality compared with those with SLN‐negative melanoma [12].

Although SLN‐negative melanoma is generally associated with a more favorable prognosis, a non‐negligible proportion of patients with SLN‐negative melanoma still develop disease recurrence, whereas data on recurrence patterns and prognostic factors in SLN‐negative patients remain limited, particularly in Asian populations where acral melanoma predominates [13, 14].

Therefore, this study aimed to characterize recurrence patterns and survival outcomes in SLN‐negative melanoma and to identify clinicopathologic factors associated with recurrence and survival in a Taiwanese cohort.

## 2 Methods

### 2.1 Study Design and Patients

This study was approved by the Institutional Review Board of Taipei Veterans General Hospital (ethics board numbers 2022‐06‐007A and 2025‐04‐014AC). We conducted a retrospective review of consecutive patients with cutaneous melanoma evaluated at Taipei Veterans General Hospital between 2005 and 2025, a tertiary medical center in Taiwan. Patients underwent wide local excision and SLNB according to standard staging protocols for clinically node‐negative melanoma.

Patients with histologically confirmed melanoma, documented SLN results, and available follow‐up data were included. Patients with stage IV disease at initial diagnosis or incomplete medical records were sexcluded. Patients were first categorized into SLN‐positive and SLN‐negative groups for overall survival comparison. Subsequent analyses of recurrence patterns and prognostic factors were restricted to the SLN‐negative cohort.

Sentinel lymph node specimens were entirely embedded for histopathological examination according to institutional protocols. Routine hematoxylin and eosin (H&E) staining was performed, and immunohistochemical staining, including Melan‐A, S100 protein, and HMB‐45, was used when clinically indicated to improve detection of micrometastatic disease.

We collected clinicopathologic variables for SLN‐negative patients, including patient‐related factors such as age, sex, body mass index (BMI), diabetes mellitus, smoking history, alcohol consumption, and betel nut use. Tumor‐related factors included Breslow thickness, ulceration, mitotic rate, lymphovascular invasion, and melanoma subtype (acral melanoma [AM] or non‐acral melanoma). AM was defined as melanoma arising from the palms, soles, or nail apparatus. Serum lactate dehydrogenase (LDH), a biomarker associated with tumor burden, was also recorded.

### 2.2 Postoperative Follow‐Up Strategy

Patients were generally followed every 3 months during the first 2 years after surgery and every 6 months thereafter. Follow‐up evaluations included physical examination, with particular attention to regional lymph node basins. Imaging studies, including ultrasonography, computed tomography (CT), positron emission tomography/computed tomography (PET/CT), or magnetic resonance imaging (MRI), were performed when clinically indicated and at the discretion of the treating physicians.

### 2.3 Outcomes

The primary outcome was recurrence‐free survival (RFS), defined as the time from definitive surgical treatment to the first documented recurrence, including local, nodal, or distant metastasis. Secondary outcomes included overall survival (OS) and patterns of first recurrence. Recurrence patterns were categorized as local or in‐transit, nodal, or distant metastasis. Patients without recurrence were censored at the date of last follow‐up.

### 2.4 Statistical Analysis

Descriptive statistics were calculated for baseline clinicopathologic variables. Categorical variables were compared using the chi‐squared test or Fisher's exact test, as appropriate. Continuous variables were analyzed using the two‐sample *t*‐test or the Mann–Whitney *U* test, depending on the distribution. Normally distributed continuous variables were presented as mean ± standard deviation, whereas skewed variables were summarized as median (interquartile range).

Survival curves for RFS and OS were estimated using the Kaplan–Meier method and compared using the log‐rank test. Cumulative incidence functions for first recurrence patterns were estimated using the Aalen–Johansen estimator under a competing‐risk framework. Cox proportional hazards regression models were used to identify predictors of RFS and OS in the SLN‐negative cohort. Variables with *p* < 0.10 in univariate analysis or those considered clinically relevant were entered into multivariate models.

Additional sensitivity analyses incorporating adjuvant anti–PD‐1 therapy were performed to assess the potential influence of evolving treatment strategies during the study period.

All statistical tests were two‐sided, and a *p*‐value < 0.05 was considered statistically significant. Statistical analyses were performed using SPSS version 31 (IBM Corp., Armonk, NY), Microsoft Excel 2021 (Microsoft Corp., Redmond, WA, USA), and R software version 4.5.3 (R Foundation for Statistical Computing, Vienna, Austria).

## 3 Results

### 3.1 Cohort Characteristics

A total of 181 patients were included in the analysis, comprising 89 patients in the SLN‐positive group and 92 in the SLN‐negative group.

**FIGURE 1:**
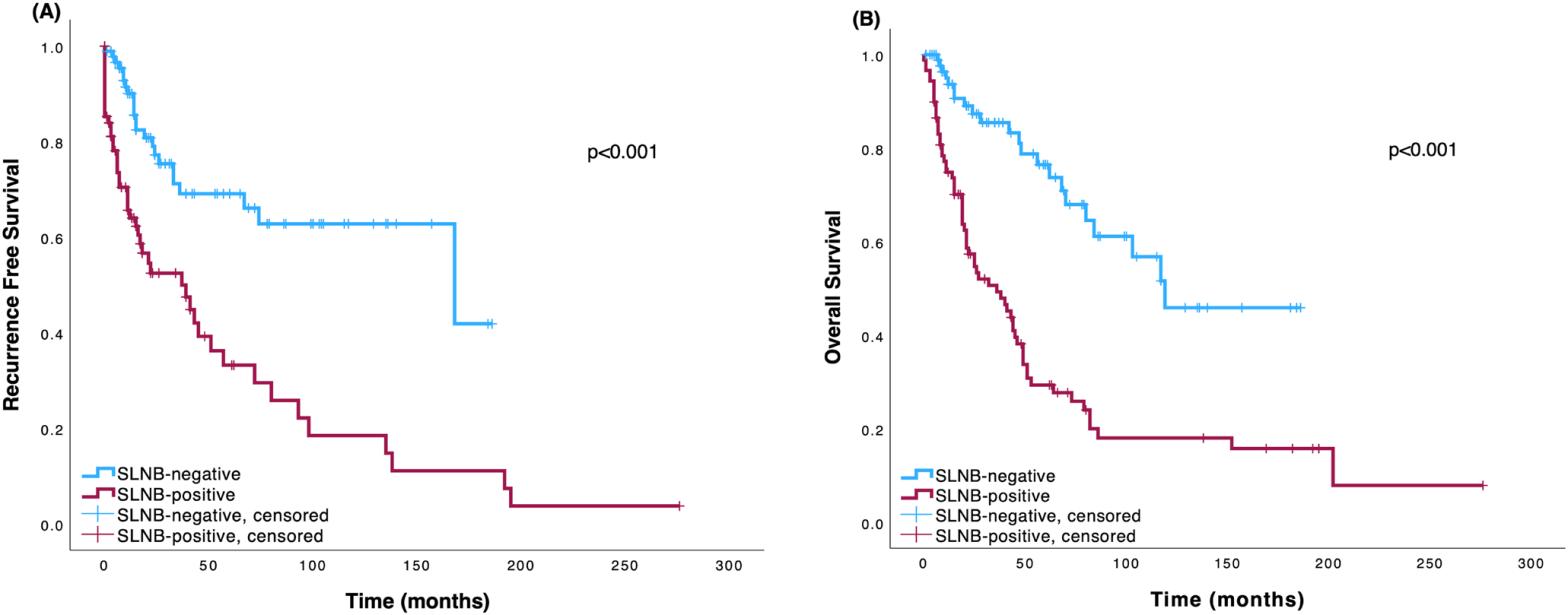
Kaplan–Meier curves of recurrence‐free survival (RFS) (A) and overall survival (OS) (B) comparing sentinel lymph node (SLN)‐positive and SLN‐negative patients. *p* values were calculated using the log‐rank test. SLNB, Sentinel lymph node biopsy.

Kaplan–Meier analysis demonstrated significantly poorer outcomes in the SLN‐positive group compared with the SLN‐negative group (Figure 1). Recurrence‐free survival (RFS) was significantly lower in SLN‐positive patients (Figure 1A, *p* < 0.001), with overall survival (OS) also significantly worse (Figure 1B, *p* < 0.001).

The SLN‐negative cohort (*n* = 92) was included in the main analysis. The mean age was 72.3 ± 11.6 years, and 48 (52.2%) were male. Acral melanoma (AM) accounted for 81 (88.0%) of cases. Baseline demographic and clinicopathologic characteristics are summarized in Table 1.

**TABLE 1:** Clinicopathologic characteristics of all SLN‐negative melanoma patients.

Patient factors	Characteristics	Patients (*n* = 92)
Sex	Male	48 (52.2%)
	Female	44 (47.8%)
Age	Mean ± SD	72.3 ± 11.6
BMI	Mean ± SD	24.5 ± 3.6
DM	Yes	4 (4.3%)
	No	81 (88.0%)
	Missing	7 (7.6%)
Smoke	Yes	12 (13.0%)
	No	79 (85.9%)
	Missing	1 (1.1%)
Alcohol	Yes	2 (2.2%)
	No	89 (96.7%)
	Missing	1 (1.1%)
Betel nut	Yes	2 (2.2%)
	No	89 (96.7%)
	Missing	1 (1.1%)

Tumor factors	Characteristics	Patients (*n* = 92)
Type	Acral melanoma	81 (88.0%)
	Non‐acral melanoma	11 (12.0%)
Breslow thickness	Median (IQR)	2.3 (1.0–4.0)
Breslow thickness	T1 (< 1 mm)	21 (22.8%)
	T2 (1–2 mm)	23 (25.0%)
	T3 (2–4 mm)	27 (29.3%)
	T4 (> 4 mm)	19 (20.7%)
	Missing	2 (2.2%)
Mitotic rate	Median (IQR)	3.0 (1.7–4.3)
LVI	Yes	0 (0%)
	No	90 (97.8%)
	Missing	2 (2.2%)
Ulceration	Yes	40 (43.5%)
	No	52 (56.5%)
Satellite	Yes	4 (4.3%)
	No	87 (94.6%)
	Missing	1 (1.1%)

Others	Characteristics	Patients (*n* = 92)
Mean LDH level	Median (IQR)	216.0 (173.8–244.0)

Abbreviations: BMI, body mass index; DM, diabetes mellitus; IQR, interquartile range; LDH, lactate dehydrogenase; LVI, lymphovascular invasion; SD, standard deviation.

### 3.2 Recurrence Patterns and Survival in SLN‐Negative Patients

**FIGURE 2:**
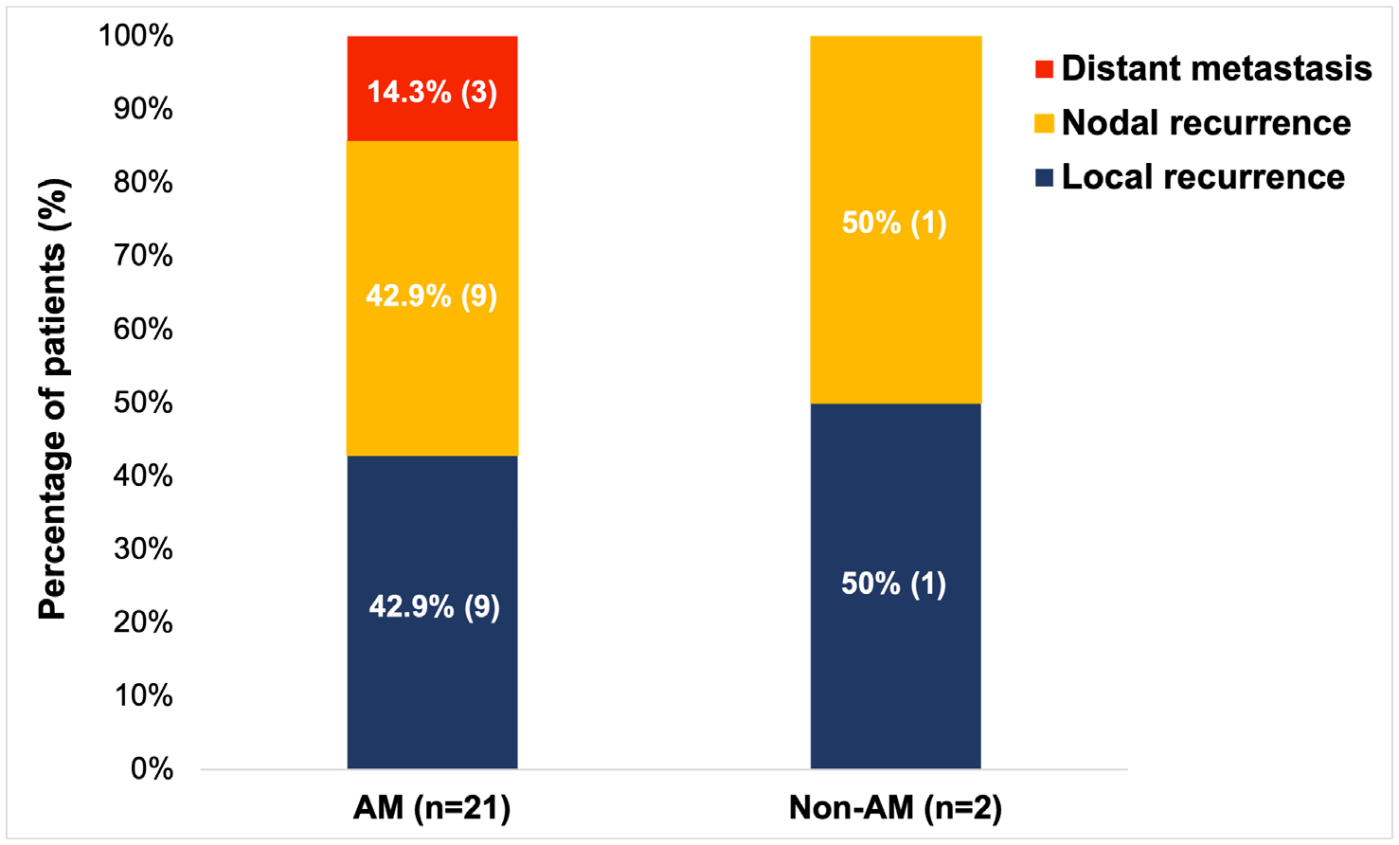
Distribution of first recurrence patterns (local, nodal, and distant metastasis) among recurrent sentinel lymph node (SLN)‐negative melanoma patients, stratified by melanoma subtype (acral vs. non‐acral melanoma). Group comparisons were performed using Fisher's exact test (*p* = 1.00). AM, acral melanoma.

During a median follow‐up of 28.5 months (interquartile range, 12.0–71.5 months), recurrence occurred in 23 patients (25.0%). Among these patients, the first recurrence was local in 10 patients (43.5%), nodal in 10 patients (43.5%), and distant in 3 patients (13.0%) (Figure 2). The 5‐year recurrence‐free survival rate was 69.1%, corresponding to a 5‐year recurrence rate of 30.9%.

**FIGURE 3:**
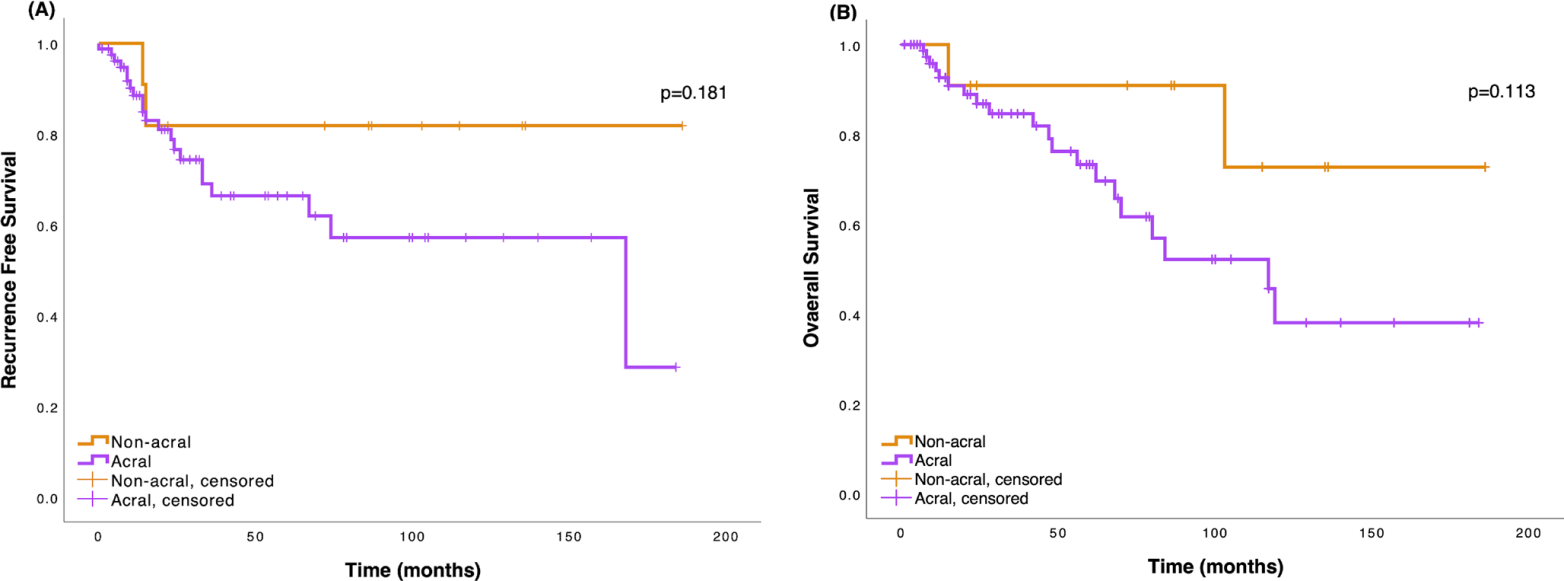
Kaplan–Meier curves of recurrence‐free survival (RFS) (A) and overall survival (OS) (B) among sentinel lymph node (SLN)‐negative melanoma patients according to melanoma subtype (acral vs. non‐acral). *p* values were calculated using the log‐rank test.

Among SLN‐negative patients, survival outcomes were further analyzed according to melanoma subtype (AM vs. non‐AM). There were no significant differences in RFS (log‐rank *p* = 0.181) or OS (log‐rank *p* = 0.113) between the two groups, although AM showed a trend toward poorer outcomes (Figure 3A,B).

**FIGURE 4:**
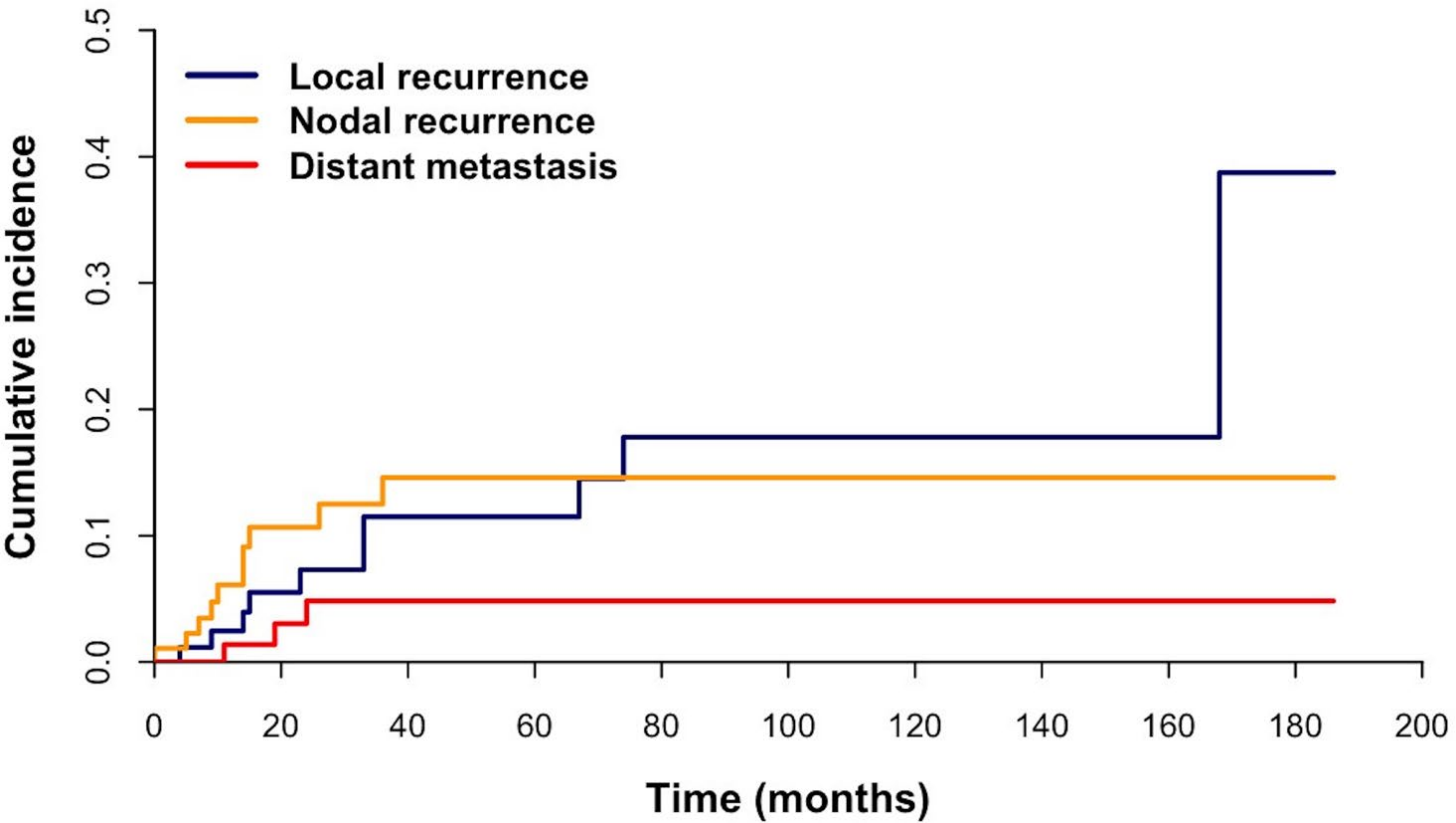
Cumulative incidence curves of first recurrence patterns (local, nodal, and distant metastasis) among all sentinel lymph node (SLN)‐negative melanoma patients.

Cumulative incidence analysis of first recurrence patterns illustrated an early accumulation of nodal recurrence events, particularly within the first year of follow‐up. At 1 year, the cumulative incidence rates were 6.1% for nodal recurrence, 2.5% for local recurrence, and 1.4% for distant metastasis. At 5 years, the corresponding rates were 14.6%, 11.5%, and 4.8%, respectively (Figure 4).

### 3.3 Cox Regression Analysis

Cox regression analysis was performed to identify factors associated with RFS and OS. In multivariate analysis, lower body mass index (BMI), diabetes mellitus (DM), and ulceration were identified as independent predictors of recurrence. Lower BMI was associated with an increased risk of recurrence (HR 0.86, 95% CI 0.75–0.97, *p* = 0.017), while DM (HR 7.59, 95% CI 1.97–29.22, *p* = 0.003) and ulceration (HR 3.29, 95% CI 1.11–9.72, *p* = 0.032) were also significantly associated with worse RFS (Table 2).

**TABLE 2:** Cox regression analysis for recurrence‐free survival in SLN‐negative melanoma patients.

Characteristic	Univariate cox		Multivariate cox	
Patient factors	HR (95% CI)	*p*	HR (95% CI)	*p*
Male (ref: Female)	0.77 (0.33–1.78)	0.535	—	—
Age	1.02 (0.99–1.05)	0.277	0.99 (0.96–1.02)	0.601
**BMI**	**0.86 (0.76–0.96)**	**0.009**	**0.86 (0.75–0.97)**	**0.017**
**DM**	**6.18 (2.01–19.02)**	**0.001**	**7.59 (1.97–29.22)**	**0.003**
Smoke	2.15 (0.84–5.51)	0.111	—	—
Alcohol	1.24 (0.17–9.51)	0.813	—	—
**Tumor factors**	**HR (95% CI)**	***p***	**HR (95% CI)**	***p***
AM (ref: Non‐AM)	2.6 (0.60–11.22)	0.200		
Breslow thickness	1.12 (0.96–1.30)	0.161	1.08 (0.85–1.39)	0.526
Mitotic rate	1.03 (0.86–1.23)	0.737	—	—
**Presence of ulceration**	**2.41 (1.04–5.59)**	**0.041**	**3.29 (1.11–9.72)**	**0.032**
**Others**	**HR (95% CI)**	***p***	**HR (95% CI)**	***p***
Mean LDH level	1.00 (0.99–1.01)	0.365	—	—

*Note:* BMI: HR per 1 kg/m^2^ increase; Breslow: HR per 1 mm increase. Bold letters indicate statistically significant differences: *p* < 0.05.

Abbreviations: AM, acral melanoma; BMI, body mass index; CI, confidence interval; DM, diabetes mellitus; HR, hazard ratio; LDH, lactate dehydrogenase.

For OS, older age, lower BMI, and greater Breslow thickness were identified as independent prognostic factors. Older age was significantly associated with poorer survival (HR 1.06, 95% CI 1.02–1.10, *p* = 0.003), with lower BMI (HR 0.85, 95% CI 0.74–0.97, *p* = 0.020) and greater Breslow thickness (HR 1.31, 95% CI 1.06–1.62, *p* = 0.014) also significantly associated with worse OS (Table 3).

**TABLE 3:** Cox regression analysis for overall survival in SLN‐negative melanoma patients.

Characteristic	Univariate cox		Multivariate cox	
Patient factors	HR (95% CI)	*p*	HR (95% CI)	*p*
Male (ref: Female)	0.41 (0.15–1.11)	0.080	—	—
**Age**	**1.06 (1.02–1.10)**	**0.001**	**1.06 (1.02–1.10)**	**0.003**
**BMI**	**0.85 (0.75–0.95)**	**0.006**	**0.85 (0.74–0.97)**	**0.020**
DM	1.92 (0.24–15.17)	0.651	0.96 (0.11–8.67)	0.986
Smoke	1.29 (0.43–3.81)	0.651	—	—
Alcohol	0.047 (0.00–1782.33)	0.569	—	—
**Tumor factors**	**HR (95% CI)**	***p***	**HR (95% CI)**	***p***
AM (ref: Non‐AM)	3.09 (0.71–13.37)	0.132		
**Breslow thickness**	**1.22 (1.04–1.43)**	**0.015**	**1.31 (1.06–1.62)**	**0.014**
Mitotic rate	1.02 (0.87–1.20)	0.821	—	—
Presence of ulceration	**2.64 (1.11–6.31)**	**0.029**	1.49 (0.55–4.03)	0.436
**Others**	**HR (95% CI)**	***p***	**HR (95% CI)**	***p***
Mean LDH level	1.00 (0.99–1.01)	0.867	—	—

*Note:* BMI: HR per 1 kg/m^2^ increase; Breslow: HR per 1 mm increase. Bold letters indicate statistically significant differences: *p* < 0.05.

Abbreviations: AM, acral melanoma; BMI, body mass index; CI, confidence interval; DM, diabetes mellitus; HR, hazard ratio; LDH, lactate dehydrogenase.

Additional sensitivity analyses incorporating adjuvant anti–PD‐1 therapy yielded generally consistent findings for recurrence‐free survival. Lower BMI and diabetes mellitus remained significantly associated with recurrence, whereas the association with ulceration was attenuated after adjustment for adjuvant anti–PD‐1 therapy (Table S1).

## 4 Discussion

In the overall cohort, SLN‐positive patients had significantly poorer outcomes, consistent with the established prognostic significance of nodal involvement. Notably, recurrence was observed in 25% of patients in our SLN‐negative cohort, exceeding the 10%–16% rates reported in predominantly non‐acral Western populations [13, 14, 15]. This difference may, in part, be attributable to the predominance of acral melanoma in our cohort. Acral melanoma is more prevalent in Asian populations and has been associated with poorer outcomes compared with non‐acral subtypes [2, 16, 17, 18, 19, 20, 21]. In our study, acral melanoma comprised 88.0% of cases and showed a numerically higher recurrence rate than non‐acral melanoma (25.9% vs. 18.2%), although this did not reach statistical significance. These findings also suggest that risk profiles derived from predominantly Western cohorts may not be directly generalizable to acral‐predominant Asian populations.

In our study, nodal recurrence was the most common first event and tended to occur early, particularly within the first year, according to cumulative incidence analysis. This pattern differs from that observed in Western cohorts, where local/in‐transit and distant metastases more frequently represent the initial site of relapse [13]. These findings suggest that nodal relapse remains an important recurrence pathway even in patients with negative SLN, particularly in acral‐predominant Asian populations.

With regard to prognostic factors, prior studies have emphasized the importance of risk stratification in SLN‐negative melanoma, including the development of nomograms incorporating ulceration, Breslow thickness, and microsatellitosis [15, 22, 23] These variables are well recognized in melanoma prognosis and are already incorporated into current staging systems, with most evidence derived from Western cohorts. In contrast, our study identified additional host‐related factors, particularly diabetes mellitus (DM) and body mass index (BMI), with independent prognostic value in an acral‐predominant Asian population.

Diabetes mellitus emerged as an independent predictor of recurrence in our cohort, although no significant association with overall survival was observed. Prior studies have shown that insulin‐dependent type 2 diabetes mellitus (T2DM) is associated with increased recurrence risk without a corresponding impact on disease‐specific survival, while population‐based data have similarly demonstrated elevated recurrence risk in male patients with localized melanoma and T2DM [24, 25]. Potential mechanisms may include chronic inflammation, impaired immune surveillance, and metabolic alterations within the tumor microenvironment [26, 27].

Our study also found that lower BMI was independently associated with both recurrence and overall survival. This finding is consistent with the “obesity paradox” described in melanoma, particularly in advanced disease treated with systemic therapies [28], although evidence across disease stages remains heterogeneous [29]. More recent evidence, including an Asian cohort reported by Chu et al., has shown improved survival among overweight patients across stages [30] Our findings extend this observation to patients with early‐stage, SLN‐negative melanoma, suggesting that BMI‐related effects may not be limited to advanced disease. However, BMI should not be interpreted as inherently protective, and the underlying mechanisms remain incompletely understood. Lower BMI may partly reflect frailty or unfavorable metabolic status associated with poorer outcomes.

Taken together, these findings highlight the potential importance of host‐related metabolic factors in the prognostic stratification of SLN‐negative melanoma. Emerging data indicate that the metabolic milieu interacts with melanoma prognosis, with melanoma patients treated with immune checkpoint inhibitors who are overweight but do not have T2DM exhibiting the most favorable outcomes [31]. In our cohort, the coexistence of lower BMI and diabetes as risk factors for recurrence highlights that host metabolic status provides an additional layer of prognostic information beyond traditional tumor‐related factors, emphasizing its potential role in risk stratification even in early‐stage, SLN‐negative melanoma. Importantly, in sensitivity analyses incorporating adjuvant anti–PD‐1 therapy, the associations between host‐related factors (BMI and diabetes mellitus) and recurrence remained consistent. These findings suggest that the observed prognostic effects are unlikely to be solely explained by treatment‐era differences and provide additional support for the robustness of our results. Combined with the observed recurrence pattern, our findings may help identify SLN‐negative patients who could benefit from closer surveillance during the early follow‐up period.

Current follow‐up guidelines for SLN‐negative melanoma generally recommend periodic clinical examination with less intensive imaging compared with node‐positive disease. However, our findings suggest that closer nodal surveillance, such as regional ultrasonography or imaging, may be considered in patients with high‐risk features, particularly during the early follow‐up period. Nevertheless, the optimal strategy for risk stratification and surveillance intensity remains to be defined, and further studies with larger cohorts are needed to validate these observations.

## 5 Limitations

Our study has several limitations. As a retrospective, single‐center analysis, the generalizability of our findings may be limited. The relatively small sample size may have reduced statistical power, particularly for subgroup analyses. In addition, the median follow‐up duration was relatively short, and late recurrence events may have been underestimated. Moreover, the study period spanned two decades during which melanoma management evolved substantially, including the introduction of adjuvant anti–PD‐1 therapy for high‐risk stage II disease. Although only a limited proportion of patients received adjuvant systemic therapy, treatment‐era effects and treatment‐selection bias may have influenced recurrence outcomes. Larger, multi‐center studies are needed to validate these findings.

## 6 Conclusion

SLN‐negative melanoma in this acral‐predominant Asian cohort was associated with a substantial risk of recurrence, with nodal relapse occurring early and frequently. Lower BMI, diabetes mellitus, and ulceration identified patients at higher risk of recurrence, while older age, lower BMI, and greater Breslow thickness predicted worse survival. These findings underscore the need for risk‐adapted surveillance strategies even in patients with negative SLN.

## Funding

This study was supported by Taipei Veterans General Hospital (V115C‐013), the National Science and Technology Council, Taiwan (NSTC 114–2314‐B‐075‐049), Y. L. LIN HUNG TAI Education and Culture Charity Trust and Y. L. LIN HUNG TAI Education Foundation, and the Melissa Lee Cancer Foundation (MLCF_V115_B11512).

## Conflicts of Interest

The authors declare no conflicts of interest.

## Supporting information


**Table S1:** Sensitivity analysis incorporating adjuvant anti–PD‐1 therapy in Cox regression models for recurrence‐free survival in SLN‐negative melanoma patients.

## Data Availability

The data supporting the findings of this study are available from the corresponding author upon reasonable request.
