# Supplementary material for: Recurrence Patterns and Prognostic Factors in Sentinel Lymph Node‐Negative Melanoma in a Taiwanese Cohort With Predominant Acral Melanoma
Source: J Dermatol. 2026 Jun 17;53(9):1236–44. doi: 10.1111/1346-8138.70358 (PMC13555145; doi:10.1111/1346-8138.70358)
Supplement: Supplementary file 1 [file JDE-53-1236-s001.docx]

**Supplementary Table 1.** Sensitivity analysis incorporating adjuvant anti–PD-1 therapy in Cox regression models for recurrence-free survival in SLN-negative melanoma patients

| Characteristic | Univariate Cox |  | Multivariate Cox |  |
| --- | --- | --- | --- | --- |
| Patient factors | HR (95% CI) | P value | HR (95% CI) | P value |
| Male (ref: Female) | 0.77 (0.33-1.78) | 0.535 | - | - |
| Age | 1.02 (0.99-1.05) | 0.277 | 1.00 (0.96-1.03) | 0.766 |
| **BMI** | **0.86 (0.76-0.96)** | **0.009** | **0.86 (0.75-0.98)** | **0.021** |
| **DM** | **6.18 (2.01-19.02)** | **0.001** | **9.34 (2.24-38.92)** | **0.002** |
| Smoke | 2.15 (0.84-5.51) | 0.111 | - | - |
| Alcohol | 1.24 (0.17-9.51) | 0.813 | - | - |
| Tumor factors | HR (95% CI) | P value | HR (95% CI) | P value |
| AM (ref: Non-AM) | 2.6 (0.60-11.22) | 0.200 |  |  |
| Breslow thickness | 1.12 (0.96-1.30) | 0.161 | 1.20 (0.98-1.46) | 0.084 |
| Mitotic rate | 1.03 (0.86-1.23) | 0.737 | - | - |
| Presence of ulceration | 2.41 (1.04-5.59) | 0.041 | 2.29 (.75-6.96) | 0.144 |
| Others | HR (95% CI) | P value | HR (95% CI) | P value |
| Mean LDH level | 1.00 (0.99-1.01) | 0.365 | - | - |
| **Anti–PD-1 therapy** | **6.10 (2.44-15.27)** | **< 0.001** | **6.642 (2.09-21.06)** | **0.001** |

**Notes:** ^†^BMI: HR per 1kg/m^2^ increase; Breslow: HR per 1mm increase. ^‡^Bold letters indicate statistically significant differences: *p* < 0.05.

**Abbreviations:** HR, Hazard ratio; CI, confidence interval; BMI, Body mass index; DM: diabetes mellitus; AM: acral melanoma

; LDH: lactate dehydrogenase
